# Functionality, physical activity, fatigue and quality of life in patients with acute COVID-19 and Long COVID infection

**DOI:** 10.1038/s41598-023-47218-1

**Published:** 2023-11-14

**Authors:** Rodrigo Vélez-Santamaría, Jessica Fernández-Solana, Fátima Méndez-López, Marta Domínguez-García, Jerónimo J. González-Bernal, Rosa Magallón-Botaya, Bárbara Oliván-Blázquez, Josefa González-Santos, Mirian Santamaría-Peláez

**Affiliations:** 1 Department of Health Sciences, University of Burgos, 09001 Burgos, Burgos Spain; 2 Primary Care Research Group, Aragon Health Research Institute (IISA), Zaragoza, Spain; 3 Network for Research on Chronicity, Primary Care, and Health Promotion (RICAPPS), Barcelona, Spain; 4 Aragonese Healthcare Service (SALUD), Zaragoza, Spain; 5 Department of Medicine, Psychiatry and Dermatology, Faculty of Medicine, University of Zaragoza, Zaragoza, Spain; 6 Department of Psychology and Sociology, University of Zaragoza, Zaragoza, Spain

**Keywords:** Health care, Diseases, Infectious diseases

## Abstract

A prominent feature of COVID-19, both in the short and long term, is the reduction in quality of life (QoL) due to low functionality scores and the presence of fatigue, which can hinder daily activities. The main objective of this study is to compare the functional status, level of physical activity, fatigue, and QoL of patients with Long COVID to other COVID-19 patients who did not develop persistent illness, and to determine whether there is a relationship between these variables and QoL. A cross-sectional study was conducted with 170 participants who had been infected with COVID-19 or had developed Long COVID. The main variables studied were functionality, physical activity, QoL and fatigue, measured using the PostCOVID-19 Functional Status Scale (PCFS), International Physical Activity Questionnaire (IPAQ), Short Form 12 (SF-12), and Fatigue Severity Scale (FSS). The main findings show a significant relationship (*p* < 0.001) between reduced functionality, lower physical activity levels, increased fatigue severity, and poorer QoL in Long COVID patients. Furthermore, these variables are also related to worse QoL, but only functional status predicts it. In conclusion, our results have shown highly significant correlations between the group with COVID-19 and Long COVID regarding functional status, level of physical activity, QoL, and fatigue.

## Introduction

The COVID-19 pandemic has triggered a global crisis that has impacted not only the realm of health but also society and the global economy. In response to this crisis, the European Union promoted cooperation among its member countries and emphasized the significance of vaccination as one of the fundamental strategies to combat the spread of the infection^1^. In the case of Spain, as of October 14, 2022, a total of 3,462,593 confirmed cases of COVID-19 have been recorded. This figure illustrates the significant impact that the pandemic has had on the country, underscoring the importance of the prevention and control measures implemented to mitigate its spread^2^.

At the beginning of 2021, over 93 million people worldwide had been infected with the SARS-CoV-2 virus. While the vast majority of individuals manage to recover from the acute infection after a few weeks, some continue to experience symptoms. Regardless of age or underlying health conditions, if these symptoms persist, it is referred to as Long COVID^3,4^. Recently, the World Health Organization (WHO) has established by international Delphi consensus the definition of Long COVID as follows: "Persistent COVID is the condition that occurs in individuals with a history of probable or confirmed SARS-CoV-2 infection, typically 3 months after onset, with symptoms lasting at least 2 months and cannot be explained by an alternative diagnosis. Common symptoms include, among others, fatigue, difficulty breathing, and cognitive dysfunction, and they generally have an impact on daily functioning. Symptoms may be newly onset after the initial recovery from an acute episode of COVID-19 or persist from the initial illness. Symptoms can also fluctuate or relapse over time"^5^.

This new condition appears to be a disease associated with several disorders, including cardiovascular, respiratory, neurological, gastrointestinal, musculoskeletal, and immunological issues, among others^6,7^. These symptoms persist for at least 12 weeks or longer and affect 10% of people who have tested positive for COVID-19. Additionally, in 70% of people who test positive, signs of deterioration in one or more organs are observed four months after the onset of symptoms^8^.

The mechanisms underlying this disease are still unknown, but it is known that persistent inflammation is a key mediator of the multifactorial genesis of long-term sequelae^9^. Some of the most frequent symptoms reported by patients with Long COVID include tiredness or fatigue, muscle pain, general malaise, dyspnea, and joint pain, among others. These symptoms may be new or continue from the initial illness^4^. Prolonged symptoms can affect the daily functioning, work, social, and domestic life of those who suffer from them^10^. This constant need for rest and repose results in a subsequent reduction in patients' daily physical activity (PA). This entails a decrease in their physical capacity and an increase in dyspnea and fatigue^11,12^. Therefore, part of the general symptomatology of these patients could be related to physical deconditioning and a reduction in exercise capacity^13^. It should be noted that these disorders can cause prolonged immobility and a reduction in PA, as well as significant limitations in functionality and a decrease in the quality of life (QoL) of patients with persistent COVID^14–16^.

Thus, in terms of functionality, COVID-19, particularly persistent COVID, is having a significant impact on people's ability to perform their Activities of Daily Living (ADLs) and maintain an active lifestyle^17^. Many individuals have experienced specific physical limitations due to the disease or resulting debilitating symptoms, affecting their ability to carry out everyday tasks, maintain employment, or engage in recreational activities^13,18,19^.

Another characteristic of COVID-19, both in the short and long term, is the decrease in health-related quality of life (QoL), directly associated with low functionality scores, as well as the presence of fatigue^17^. Persistent fatigue is also the most common symptom in Long COVID patients regardless of the severity of the initial presentation or the presence of respiratory difficulty^20^. It is a persistent feeling of tiredness or exhaustion that cannot be alleviated with rest and it is also not proportional to levels of physical activity. Chronic fatigue also encompasses multiple dysfunctions that interfere with functioning and therefore negatively impact QoL^21^. Several studies have observed a reasonable similarity between perceived fatigue in Long COVID and Chronic Fatigue Syndrome (CFS)^22^. The presence of chronic fatigue as a symptom may be attributed to damage in multiple organ systems, including the cardiac, pulmonary, and renal systems, resulting from COVID-19 infection^23^. This is why part of the general symptomatology of these patients could be related to physical deconditioning and reduced exercise capacity^13^.

Thus, various studies have also reported a strong association between COVID-19 and QoL between 6 and 12 months after acute infection^24,25^. Within the context of QoL, both physical and emotional/social components of a patient's experience are encompassed^26^. The physical aspect of QoL is, in turn, related to the functional status, independence, and degree of participation in Activities of Daily Living (ADLs)^27^. Therefore, adding the concept of QoL is considered necessary, as it is deemed a fundamental factor when examining the impact that diseases have on the physical, mental, and social aspects of patients' health^28^. Furthermore, it can provide essential information about the disease's impact on patients' daily lives.

Although some articles have analyzed these aspects, there is still limited evidence regarding the true impact on patients who develop Long COVID^29^. Furthermore, there is a lack of studies that investigate the relationship between functional status, the level of physical activity, or other symptoms such as fatigue and the QoL of Long COVID patients, despite their potential significance^14,15^.

Some studies conducted in hospitalized cohorts with acute COVID-19 infection have reported that a considerable number of patients experienced poor exercise capacity or exercise tolerance and moderate to severe fatigue^30^. In addition, deficiencies in functional mobility were identified along with long-term sequelae from COVID-19 and impaired performance in ADLs^15^. Several months after infection, they reported a significant deterioration in their QoL compared to the pre-COVID-19 infection state^14^. Although several studies have assessed the symptom burden of Long COVID patients in comparison to those with acute COVID-19, few have explored the functional status in long-term patients^4,7^.

Some authors emphasize the need to obtain information on the role of physical activity in the treatment and rehabilitation of Long COVID patients due to the lack of evidence in this regard^13^. Likewise, to date, many studies have been conducted to explore persistent symptoms such as chronic fatigue and functional status in hospitalized patients, but there is still a lack of evidence in non-hospitalized cohorts with Long COVID^10^.

The aim set in this study is to compare the functional status, level of physical activity, QoL, fatigue, among patients diagnosed with Long COVID and those who were infected with COVID-19 but did not develop symptoms compatible with the diagnosis of Long COVID. Additionally, to determine if there is a relationship between these variables and QoL.

## Methods

### Study design

This was a cross-sectional, retrospective study registered in the International Traditional Medicine Clinical Trial Registry (ISRCTN) (ISRCTN27312680). The sample size was calculated using data from the Global Burden of Disease Long COVID Collaborators study. We used the prevalence of long COVID as the primary endpoint, being 10–20% of all patients who had symptomatic SARS-CoV-2 infection in 2020 and 2021. For the sample size calculation, we conducted a one-sided test with a 95% confidence level of and 80% statistical power. We made an empirically assumption that up to 30% of the analytical abnormalities might persist in the control group (Group 1) and up to 50% of them in the intervention group (considering only a 20% difference between them) (Group 2). This calculation indicated a total sample size requirement of 156 participants, with73 participants allocated to each group to detect this difference.

### Participants and procedure

This study was conducted between December 2021 and July 2022 at primary healthcare centers in Zaragoza, Aragón, located in northern Spain. During this timeframe, we collected the sample, which included individuals affiliated with the “Long COVID Aragón” support group and those who had primary care appointments (both in-person and via telephone) for any reason and met the inclusion criteria during their consultations.

Once deemed eligible by their primary care physician and provided they met the established criteria and consented to participate in the study, these individuals were referred to the study coordinators. The Aragonese Primary Care Research Group screened patients to confirm their eligibility. Inclusion criteria were individuals aged 16 or older with a diagnosis of acute COVID-19, either at the time they were referred by their primary care physician or previously, based on a diagnostic test confirming a previous COVID-19 infection. Proficiency in spoken and written Spanish was also required.

Participant data were collected after a minimum of three months had passed since their acute COVID-19 infection. After this period, participants were categorized into two groups: Group 1 consisted of individuals who, at least three months after their acute infection, were diagnosed with Post-Acute COVID-19 Syndrome (PACS) using the WHO Delphi consensus for post-COVID-19 conditions^5^, commonly referred to as Long COVID. Group 2 included patients who did not have a diagnosed PACS, did not exhibit suspected symptoms, and had only experienced acute COVID-19 at least three months prior (diagnosed through reverse transcription polymerase chain reaction [RT-PCR], rapid antigen tests, or SARS-CoV-2 serology).

Exclusion criteria included (A) the presence of symptoms before acute SARS-CoV-2 infection, (B) refusal or incapacity to provide consent or communicate, and (C) being institutionalized at the time of the appointment.

A single measurement was obtained from all participants in both groups, enabling us to collect data on variables for each participant when a minimum of three months had elapsed since their diagnosed acute COVID-19 infection.

The Bioethics Committee of the University of Burgos approved the research, (Reference UBU 032/2021), respecting all the requirements established in the Declaration of Helsinki of 1975. This research was approved by the Provincial Research Ethics Committee of Córdoba (Ref. 5033). Informed consent was obtained from all study participants.

### Variables and assessments

In the sociodemographic questionnaire, we gathered data on variables such as sex, age, marital status, employment status, usual profession, history of sick leave due to COVID-19, level of education, and the need for special care due to disability, dependence, or limitations in ADLs..

Information was obtained using the following scales to assess functional status, level of physical activity, QoL, and fatigue.

The PostCOVID-19 Functional Status Scale (PCFS), is specifically designed for post COVID-19 follow-up. This scale assesses critical aspects of daily life to identify functional limitations in patients who have had or are currently experiencing COVID-19. Its aims is to determine the impact of the infection on an individual's functional status. The scale addresses a wide range of functional outcomes, focusing on limitations in daily tasks and lifestyle changes. Functional status is rated on a scale from 0 (indicating no limitations) to the highest score, signifying more severe limitations^31^.

This questionnaire gathered information regarding the current functional status of participants who had experienced a COVID-19 infection at least three months prior to data collection.International Physical Activity Questionnaire (IPAQ), is an instrument developed to standardize the criteria for assessing physical activity. It comprises 7 questions concerning the frequency, duration, and intensity of physical activity. After completing the questionnaire, reference values are applied to classify physical activity into one of three categories: high, moderate, or low^32^.

The IPAQ questionnaire collected information at two different time points. The first IPAQ questionnaire inquired about the participants’ level of physical activity before they contracted COVID-19. The second IPAQ questionnaire, included in the overall data collection form, gathered information about the participants’ level of physical activity at the time of data collection.Short Form 12 (SF-12), is a questionnaire designed to assess health-related QoL. Comprising 12 items, its purpose is to provide an user-friendly tool for evaluating individuals' functional capacity and well-being. Itdefines both positive and negative states of physical and mental health through 8 dimensions (physical functioning, role physical, bodily pain, mental health, general health, vitality, social functioning, and role emotional). Scores on the SF-12 range from 0 to 100, with higher scores indicating better QoL. It is a valid and reliable measure with internal consistency estimates exceeding 0.70^33^.

Similar to the previous questionnaire, the SF-12 collected information about participants' pre-COVID-19 Quality of Life (QoL). Another SF-12 questionnaire, included in the data collection form, gathered information about participants' current QoL at the time of data collection.Fatigue Severity Scale (FSS), consists of 9 Likert-type items with 7 increasing intensity responses, scored from 1 to 7. It demonstrates an internal consistency of 0.88^34^.

For the FSS as well, information was collected both before the COVID-19 infection and at the current time of data collection.

Criteria for CFS. For the diagnosis of CFS, several criteria are used to delineate CFS and contribute to a better understanding of its possible solutions. These criteria are: (A) chronic persistent (at least 6 months), or intermittent, unexplained fatigue that is new or with definite onset and is not the result of recent exertion, does not improve with rest and results in a marked reduction in the patient's previous usual activity; (B) exclusion of other illnesses that may be a cause of chronic fatigue, concurrently, 4 or more of the following minor criteria (signs or symptoms) must be present, all persistent for 6 months or more and subsequent to the presence of fatigue: (1) recent impaired concentration or memory; (2) odynophagia; (3) painful cervical or axillary lymphadenopathy; (4) myalgia; (5) polyarthralgia without signs of phlogosis; (6) headache of recent onset or of different characteristics than usual; (7) non-refreshing sleep; (8) post-exertional malaise lasting more than 24 h^35^.

In the following Table 1, the data collection questionnaires are presented along with the respective variables they measure for participants in this study.

**Table 1:** Assessment and Variables.

Assessments	Variables
PCFS	Functional status post-COVID-19
IPAQ	Level of physical activity
SF-12	QoL
FSS	Level of fatigue
Critaria for CFS	CFS

### Statistical analysis

The data were collected virtually through a questionnaire in Google Drive, which was later exported to an Excel spreadsheet and statistically analyzed using the SPSS version 25.0 software (IBM-Inc., Chicago, IL, USA).

First, a descriptive analysis of the sample characteristics was carried out, expressing categorical variables as absolute frequencies and percentages, and continuous variables as means and standard deviations (SD).

Next, an analysis was performed using chi-square tests and t-tests for independent samples between the different variables to be analyzed and the group, as well as some of the variables and quality of life; differences were considered statistically significant for a value of *p* ≤ 0.05. To analyze the factors associated with quality of life, a stepwise regression was used with the variables that showed significant results in the previous analysis, using these variables as independent variables and quality of life as the dependent variable.

## Results

The demographic and descriptive characteristics of the 170 participants who took part in the study are presented in Table 2, along with the characteristics of each of the study groups, i.e., the group diagnosed with Long COVID or PACS (n = 85) and the second group of participants who had only experienced an acute COVID-19 infection at least three months prior to data collection and did not exhibit any symptoms compatible with the diagnosis of Long COVID or PACS (n = 85).

**Table 2:** Demographic and descriptive characteristics of the sample.

Variables	Without Long COVID or PACS (Group 1)	With Long COVID or PACS (Group 2)	Total (n = 170)	*p*-value
Age	48.12 ± 9.70	47.33 ± 10.00	47.72 ± 9.83	0.603
Group	85 (50%)	85 (50%)	170 (100%)	
Sex				
Men	19 (22.4%)	17 (20%)	36 (21.2%)	0.707
Women	66 (77.6%)	68 (80%)	134 (78.8%	
Marital Status				
Single	11 (12.9%)	12 (14.1%)	23 (13.5%)	0.728
Married/living with a partner	64 (75.3%)	60 (70.6%)	124 (72.9%)	
Separated/divorced	8 (9.4%)	12 (14.1%)	20 (11.8%)	
Widowed	2 (2.4%)	1 (1.2%)	3 (1.8%)	
Employment Status				
Active	69 (81.2%)	33 (38.8%)	102 (60%)	< 0.001
Leave of absence > 3 months	2 (2.4%)	39 (45.9%)	41 (24.1%)	
Retired	6 (7.1%)	5 (5.9%)	11 (6.5%)	
Other	8 (9.4%)	8 (9.5%)	16 (9.5%)	
Sick leave due to COVID-19				
Yes	55 (64.7%)	73 (85.9%)	128 (75.3%)	0.002
No	19 (22.4%)	4 (4.7%)	23 (13.5%)	
Not applicable (disability, retirement…)	11 (12.9%)	8 (9.4%)	19 (11.2%)	
Leve of studies				
Without studies	–	1 (1.2%)	1 (0.6%)	0.669
Primary school	5 (5.9%)	3 (3.5%)	8 (4.7%)	
Secondary school	38 (44.7%)	37 (43.5%)	75 (44.1%)	
University or more	42 (49.4%)	44 (51.8%)	86 (50.6%)	
Requires special dedication due to dependence, disabilty or limitations for ADLs				
Yes	6 (7.1%)	12 (14.1%)	18 (10.6%)	0.109
No	79 (92.9%)	73 (86%)	150 (88.2%)	

*ADLs* Activities of Daily Living.

The mean age of the entire sample (n = 170) was 47.71 ± 9.83 years, with a lower mean age observed in the group diagnosed with Long COVID.

Statistically significant differences are observed between the two groups in terms of employment status (*p* = 0.002), with a higher percentage of the non-Long COVID population being actively employed and having been on sick leave for more than three months. Statistically significant differences are also observed in COVID-19-related sick leaves (*p* < 0.001), with a higher percentage of sick leave in the group diagnosed with Long COVID.

The Tables 3 and 4 show the relationship between the scores on the different scales used and the group. Furthermore, Supplementary Tables 1 to 9 can be observed in the supplementary material, showing expanded results of the correlation between the various variables presented in Table 3 and the groups. Significant correlations were obtained for all of them. In terms of functional status, the Long COVID group had severe functional limitations in 95% of cases, compared to the acute infection group. Prior to COVID-19 infection, the Long COVID group had a high level of physical activity (60.2%), while current physical activity is low in 79.3% of this group. This means that 44.7% of patients with Long COVID have experienced a worsening of their physical activity, while in the acute infection group, it has remained the same (84.7%). Prior to COVID, the quality of life was expressed as very good (45.9%) for the Long COVID group and good (47.1%) for the acute infection group. However, the variation in their quality of life indicates that the Long COVID group has deteriorated significantly (54.1%), while the other group has remained the same (88.2%). With respect to changes in the severity of fatigue, it has worsened significantly (94.7%) in Long COVID cases, with 92.4% meeting the criteria for CFS (Tables 3 and 4).

**Table 3:** Correlation between variables and group.

Variable	Group 1/Group 2	
	Value	*p*-value
Functional status post-COVID-19	111.57	< 0.001
Level of physical activity pre-COVID-19 and current	9.12	0.010
Current level of physical activity	15.22	< 0.001
Variation in physical activity level pre COVID and current	52.76	< 0.001
QoL pre COVID-19	9.80	0.020
Current QoL	91.00	< 0.001
Variation in QoL pre COVID and current	135.87	< 0.001
Variation in level of fatigue pre COVID and current	93.15	< 0.001
SFC (compatible yes/no)	108.19	< 0.001

*PCFS* PostCOVID-19 Functional Status Scale, *IPAQ* International Physical Activity Questionnaire, *SF-12* Short Form 12, *FSS* Fatigue Severity Scale, *CFS* Chronic Fatigue Syndrome.

**Table 4:** T-test between variables and group.

Variable	Group			
	Mean	Sd	t	*p*-value (Bilateral)
Current level of fatigue	52.28	14.29	13.89	0.066
Possible criteria for diagnosis of CFS	5.56	1.64	19.60	0.031

*FSS* Fatigue Severity Scale, *CFS* Chronic Fatigue Syndrome, *Sd* Standard Deviation.

Table 5 shows the significant correlations between the variables of functional status, current physical activity, and changes in fatigue severity with respect to QoL. Functional status revealed that 75% of people without functional limitations reported excellent QoL, compared to 80% of those with severe functional limitations reporting poor QoL. Similarly, 75% of people who had no fatigue before or now had excellent QoL, while 30% of those who had worsened significantly reported poor QoL.

**Table 5:** Correlation between functional status, physical activity and fatigue-quality of life.

Variable	Current QoL	
	Value	*p*-value
Functional status post-COVID-19	104.66	< 0.001
Current level of physical activity	18.21	0.020
Variation in level of fatigue pre COVID and current	79.03	< 0.001

*PCFS* PostCOVID-19 Functional Status Scale, *IPAQ* International Physical Activity Questionnaire, *SF-12* Short Form 12, *FSS* Fatigue Severity Scale.

A stepwise regression was performed with the variables that previously had a significant relationship, namely functional status, level of physical activity, and fatigue variation. In this, a model was formed using the functional status variable (F(1, 166) = − 345.081, *p* < 0.001, R = 0.822, R2 = 0.675, corrected R2 = 0.673; functional status (β = − 0.822, *p* < 0.001)), excluding the other variables from the model.

## Discussion

The first aim of this study was to compare the functional status, level of physical activity, QoL, fatigue, among patients diagnosed with Long COVID and those who were infected with COVID-19 but did not develop symptoms compatible with the diagnosis of Long COVID.

Our results have shown positive and highly significant correlations between the group diagnosed with Long COVID and those who did not develop symptoms compatible with it, regarding functional status, physical activity level, QoL, and fatigue. It has been observed that participants diagnosed with Long COVID have demonstrated severe functional limitations in a much higher percentage than the group that did not develop symptoms compatible with this new condition. Likewise, their level of physical activity is low, and they have experienced a worsening compared to their previous state. Regarding QoL, our results have revealed a greater deterioration in those patients diagnosed with Long COVID compared to the group that did not develop symptoms following the infection, in which maintenance is observed.

Among people diagnosed with Long COVID, persistent symptoms can cause various disruptions, including dyspnea, fatigue, functional impairments, and cognitive repercussions^36^. It is understandable that these complications have significant clinical and practical relevance for health and well-being^37^. This fact also raises important questions about the functional presentation and consequences of Long COVID, especially in women, who are a majority group, as can also be seen in our sample; and since they tend to have greater age-related disability than men^38^. Similarly, despite not requiring hospitalization, people with COVID-19 infection may also be at risk for deterioration in physical, cognitive, and mental health^39^.

Furthermore, some studies support our results, finding a debilitating symptom profile in patients that reflects far-reaching consequences for health and well-being^40^. Prolonged periods of morbidity and decreased engagement in daily activities, sedentary behavior, and consequent QoL decline have been observed^41^. Long COVID has also been found to impact the energy reserves of those who suffer from it^42^.

One study found no association between the duration of COVID infection and levels of physical activity and independence. However, it was observed that individuals with a longer duration of the disease were unable to regain their previous levels of physical activity^43^. Other studies also demonstrated low levels of physical activity due to more sedentary behavior^44^.

According to our results, a study reported that more than half of the patients who developed Long COVID had a worse QoL. These patients reported pain or discomfort, health problems, mobility impairments, problems related to daily activities, or self-care^45^. Other studies have also reported that Long COVID results in poor QoL, possibly caused by post-traumatic stress syndrome^46^. However, it is most likely that the consequences of COVID-19 alone or in combination can reduce QoL^47^. In contrast, Narayan Poudel et al.^48^ concluded that acute COVID-19 infection had a greater impact on QoL than Long COVID.

On the other hand, taking into account the severity of fatigue, our results have shown a significant worsening in those patients with Long COVID, meeting CFS criteria in 92.4% of cases. Another study also found CFS in over half of their sample, with a significant burden of fatigue at a median follow-up of 10 weeks^6^. This is especially concerning, given that it is recommended that returning to work after a viral infection should occur after 4 weeks to avoid deconditioning^49^. Post-COVID fatigue, according to the literature, is found in 40% of individuals one year after the initial infection, and 1 in 4 meet the diagnostic criteria for CFS at that time^6,13^. Fatigue, moreover, is not only extremely common in cases of Long COVID, but its severity and persistence can disrupt life^50^. Although it is currently unclear how to treat chronic fatigue in people with Long COVID, pulmonary rehabilitation may be key in the treatment of these individuals^13^.

As a second objective for this study, we aimed to determine whether functionality, the level of physical activity, and the severity of fatigue were related to the patients' QoL. Our results also show a highly significant correlation of these analyzed variables with the patients' QoL. This implies that those patients in the sample with more severe functional limitations, lower levels of physical activity, and greater fatigue severity have experienced a more marked decrease in their QoL. However, despite having a significant relationship, only the patients' functional status has proven to be a predictor of QoL.

As observed so far, one of the main characteristics of Long COVID is the disability generated by its symptoms, which can alter functionality to such an extent that it incapacitates the patient in the performance of everyday tasks^51^. In Spain, it has been found that more than 70% of patients diagnosed with Long COVID had limitations that disabled them in the performance of ADLs. This disability had a significant impact on their QoL^52^. Various exploratory analyses are consistent with our results regarding the relationship between fatigue, reduction of function or increase in disability, and reduction in QoL^53^.

This highlights the need to recover QoL, and for this, it has been shown that exercise provides a significant improvement in pain tolerance, movement, optimization of physical function, and recovery of autonomy in ADLs. In addition, the psychological improvements resulting from exercise could provide patients with a coping strategy to help overcome the health challenge, directly influencing their QoL^10^.

In this sense, both physical inactivity and sedentary behaviour are independent risk factors for multiple harmful health effects and a reduction in QoL^54^. Therefore, establishing rehabilitation programs to prevent the secondary consequences of physical inactivity is a priority^43^.

The range of symptoms associated with Long COVID, as well as their severity, frequency, and duration, are posing significant challenges for the publication of precise recommendations on physical activity in these individuals^42^. In one study, it was found that after 8 weeks of physical rehabilitation, physical function and exercise capacity could be improved, but functional status remained below the expected value for the patient's age^50^. In another study, it was found that after 6 weeks of pulmonary rehabilitation, exercise capacity of patients with Long COVID was significantly improved^13^.

While some limitations were found in the study, further research is needed to establish the causality of the consequences of Long COVID and its impact on daily life, as this was a cross-sectional study. The study was conducted in a single Spanish autonomous community, which could be considered a limitation when extrapolating the results to the general population. Therefore, it would be necessary to expand the sample and broaden the geographic area, including different urban and rural areas. There is a need for further research in this area, as few studies have been conducted on this topic. It is also worth noting that the type of survey used can introduce information bias as it is a self-administered survey.

In conclusion, our results have shown highly significant correlations between the group that developed Long COVID and those who experienced no symptoms regarding functional status, level of physical activity, QoL, and fatigue. We found severe functional limitation impairment, low levels of physical activity, a considerable worsening of their QoL, and fatigue in patients diagnosed with Long COVID.Likewise, functionality, the level of physical activity, and severity of fatigue are also directly related to QoL. Therefore, the worse the functional status, level of physical activity, and fatigue, the worse their QoL. Although only one of these variables has been found to be a predictor of QoL, which is the person's functional status.

The current findings highlight the urgent need for the creation of specific strategies for addressing Long COVID, and support pathways for patients that enable the recovery of the pre-COVID-19 QoL^55^. According to our results, there is also a need to address this profound physical and QoL impact through recommendations adapted to the clinical state of each individual and multidisciplinary approaches through different healthcare professionals. Physical and cognitive rehabilitation is highlighted in this case to address deficits caused as a consequence of COVID-19 infection^50^.

The importance of continuing to conduct studies that outline a correct path towards the recovery of Long COVID patients is emphasized, outlining to what extent the chronic effects of Long COVID affect the health, well-being, and QoL of the population. The growing burden on rehabilitation highlights the need to develop effective interventions and multidisciplinary support pathways.

### Supplementary Information

Supplementary Tables.

## Data Availability

The datasets generated and/or analysed during the current study are not publicly available due the participants of this study did not give written consent for their data to be shared publicly, but are available from the corresponding author on reasonable request.

## References

[1] Ministerio de Sanidad [Internet]. Ministerio de Sanidad. 2022. Available from: https://www.sanidad.gob.es/profesionales/saludPublica/ccayes/alertasActual/nCov/situacionActual.htm

[2] Consilium. COVID-19: respuesta de la UE en el ámbito de la salud pública [Internet]. Consejo Europeo. 2022. Available from: https://www.consilium.europa.eu/es/policies/coronavirus/covid-19-public-health/

[3] McCorkell L, Assaf GS, Davis HE, Wei H, Akrami A (2021). Patient-led research collaborative: Embedding patients in the Long COVID narrative. Pain Rep..

[4] Romero-Rodríguez E, Perula-de-Torres LÁ, González-Lama J, Castro-Jiménez RÁ, Jiménez-García C, Priego-Pérez C (2023). Long COVID symptomatology and associated factors in primary care patients: The EPICOVID-AP21 study. Healthcare (Basel, Switzerland).

[5] Soriano JB, Murthy S, Marshall JC, Relan P, Diaz JV (2022). A clinical case definition of post-COVID-19 condition by a Delphi consensus. Lancet Infect. Dis..

[6] Townsend L, Dyer AH, Jones K, Dunne J, Mooney A, Gaffney F (2020). Persistent fatigue following SARS-CoV-2 infection is common and independent of severity of initial infection. PloS One.

[7] Romero-Rodríguez E, Pérula-de Torres LÁ, Castro-Jiménez R, González-Lama J, Jiménez-García C, González-Bernal JJ (2022). Hospital admission and vaccination as predictive factors of long COVID-19 symptoms. Front. Med..

[8] The prevalence of long COVID symptoms and COVID-19 complications - Office for National Statistics [Internet]. [cited 2023 Jan 25]. Available from: https://www.ons.gov.uk/news/statementsandletters/theprevalenceoflongcovidsymptomsandcovid19complications

[9] Maltezou HC, Pavli A, Tsakris A (2021). Post-COVID syndrome: An insight on its pathogenesis. Vaccines.

[10] The PHOSP-COVID Collaborative Group (2022). Clinical characteristics with inflammation profiling of long COVID and association with 1-year recovery following hospitalisation in the UK: a prospective observational study. Lancet Respir. Med..

[11] Belli, S. *et al*. Low physical functioning and impaired performance of activities of daily life in COVID-19 patients who survived hospitalisation. *Eur Respir J .* **56**(4), 2002096 (2020). 10.1183/13993003.02096-2020 PMC7411272 32764112

[12] Rooney S, Webster A, Paul L (2020). Systematic review of changes and recovery in physical function and fitness after severe acute respiratory syndrome-related coronavirus infection: implications for COVID-19 rehabilitation. Phys. Ther..

[13] Besnier F, Bérubé B, Malo J, Gagnon C, Grégoire CA, Juneau M (2022). Cardiopulmonary rehabilitation in long-COVID-19 patients with persistent breathlessness and fatigue: The COVID-rehab study. Int. J. Environ. Res. Public Health.

[14] Delbressine JM, Machado FVC, Goërtz YMJ, Van Herck M, Meys R, Houben-Wilke S (2021). The impact of post-covid-19 syndrome on self-reported physical activity. Int. J. Environ. Res. Public Health.

[15] Tleyjeh IM, Saddik B, Ramakrishnan RK, AlSwaidan N, AlAnazi A, Alhazmi D (2022). Long term predictors of breathlessness, exercise intolerance, chronic fatigue and well-being in hospitalized patients with COVID-19: A cohort study with 4 months median follow-up. J. Infect. Public Health.

[16] Nasserie T, Hittle M, Goodman SN (2021). Assessment of the frequency and variety of persistent symptoms among patients with COVID-19: A systematic review. JAMA Netw. Open.

[17] Muñoz-Corona C, Gutiérrez-Canales LG, Ortiz-Ledesma C, Martínez-Navarro LJ, Macías AE, Scavo-Montes DA (2022). Quality of life and persistence of COVID-19 symptoms 90 days afterhospital discharge. J. Int. Med. Res..

[18] Meys R, Delbressine JM, Goërtz YMJ, Vaes AW, Machado FVC, Van Herck M (2020). Generic and respiratory-specific quality of life in non-hospitalized patients with COVID-19. J. Clin. Med..

[19] Arnold DT, Hamilton FW, Milne A, Morley AJ, Viner J, Attwood M (2021). Patient outcomes after hospitalisation with COVID-19 and implications for follow-up: Results from a prospective UK cohort. Thorax.

[20] Townsend L, Dowds J, O’Brien K, Sheill G, Dyer AH, O’Kelly B (2021). Persistent poor health after COVID-19 is not associated with respiratory complications or initial disease severity. Ann. Am Thorac. Soc..

[21] Penner IK, Paul F (2017). Fatigue as a symptom or comorbidity of neurological diseases. Nat. Rev. Neurol..

[22] Bansal R, Gubbi S, Koch CA (2022). COVID-19 and chronic fatigue syndrome: An endocrine perspective. J. Clin. Transl. Endocrinol..

[23] Karlsson AC, Humbert M, Buggert M (2020). The known unknowns of T cell immunity to COVID-19. Sci. Immunol..

[24] Tsuzuki S, Miyazato Y, Terada M, Morioka S, Ohmagari N, Beutels P (2022). Impact of long-COVID on health-related quality of life in Japanese COVID-19 patients. Health Qual. Life Outcomes..

[25] Shanbehzadeh S, Zanjari N, Yassin M, Yassin Z, Tavahomi M (2023). Association between long COVID, functional activity, and health-related quality of life in older adults. BMC Geriatr..

[26] Ojeda A, Calvo A, Cuñat T, Mellado-Artigas R, Comino-Trinidad O, Aliaga J (2022). Characteristics and influence on quality of life of new-onset pain in critical COVID-19 survivors. Eur. J. Pain.

[27] Hays, R. D. & Reeve, B. B. Measurement and Modeling of Health-Related Quality of Life. International Encyclopedia of Public Health. 241–52 (2008).

[28] Brettschneider C, Leicht H, Bickel H, Dahlhaus A, Fuchs A, Gensichen J (2013). Relative impact of multimorbid chronic conditions on health-related quality of life—Results from the MultiCare Cohort Study. PLOS ONE.

[29] Walle-Hansen MM, Ranhoff AH, Mellingsæter M, Wang-Hansen MS, Myrstad M (2021). Health-related quality of life, functional decline, and long-term mortality in older patients following hospitalisation due to COVID-19. BMC Geriatr..

[30] Groff D, Sun A, Ssentongo AE, Ba DM, Parsons N, Poudel GR (2021). Short-term and long-term rates of postacute sequelae of SARS-CoV-2 infection: A systematic review. JAMA Netw. Open.

[31] Lorca LA, Leão Ribeiro I, Torres-Castro R, Sacomori C, Rivera C (2022). Propiedades psicométricas de la escala Post-COVID-19 Functional Status para adultos sobrevivientes de COVID-19. Rehabilitacion.

[32] Martínez-Aldao D, Diz JC, Varela S, Ayán C (2019). Analysis of the convergent validity of the Spanish short version of the Minnesota Leisure Time Physical Activity Questionnaire (VREM) and the Spanish version of the International Physical Activity Questionnaire in elderly people (IPAQ-E). An sistema sanitario de Navarra.

[33] Vera-Villarroel P, Silva J, Celis-Atenas K, Pavez P (2014). Evaluación del cuestionario SF-12: Verificación de la utilidad de la escala salud mental. Revista médica de Chile.

[34] Bernal-Vargas L, Riveros-Munévar F, Vinaccia-Alpi S, Quiceno-Sierra J-M, Bernal-Vargas L, Riveros-Munévar F (2017). Estructura factorial y consistencia interna de la Escala de severidad de fatiga en población colombiana con enfermedades crónicas. Enferm. Glob..

[35] Avellaneda Fernández A, Pérez Martín Á, Izquierdo MM (2009). Síndrome de fatiga crónica Documento de consenso. Atención Primaria.

[36] Yan Z, Yang M, Lai CL (2021). Long COVID-19 syndrome: A comprehensive review of its effect on various organ systems and recommendation on rehabilitation plans. Biomedicines.

[37] Carter, S. J., Baranauskas, M. N., Raglin, J. S., Pescosolido, B. A. & Perry B. L. Functional status, mood state, and physical activity among women with post-acute COVID-19 syndrome. medRxiv: the preprint server for health sciences. (2022). 10.3389/ijph.2022.1604589 PMC9218064 35755951

[38] Hosseinpoor AR, Williams JS, Jann B, Kowal P, Officer A, Posarac A (2012). Social determinants of sex differences in disability among older adults: a multi-country decomposition analysis using the World Health Survey. Int. J. Equity Health.

[39] Mayer KP, Steele AK, Soper MK, Branton JD, Lusby ML, Kalema AG (2021). Physical therapy management of an individual with post-COVID syndrome: A case report. Phys. Ther..

[40] Faghy MA, Maden-Wilkinson T, Arena R, Copeland RJ, Owen R, Hodgkins H (2022). COVID-19 patients require multi-disciplinary rehabilitation approaches to address persisting symptom profiles and restore pre-COVID quality of life. Expert Rev. Respir. Med..

[41] Hayes LD, Ingram J, Sculthorpe NF (2021). More than 100 persistent symptoms of SARS-CoV-2 (Long COVID): A scoping review. Front. Med..

[42] Humphreys H, Kilby L, Kudiersky N, Copeland R (2021). Long COVID and the role of physical activity: A qualitative study. BMJ open.

[43] Wright, J., Astill, S. L. & Sivan, M. The Relationship between Physical Activity and Long COVID: A Cross-Sectional Study. *Int J Environ Res Public Health .* **19**(9), 5093 (2022). 10.3390/ijerph19095093 PMC9105041 35564488

[44] Tremblay MS, Aubert S, Barnes JD, Saunders TJ, Carson V, Latimer-Cheung AE (2017). Sedentary Behavior Research Network (SBRN)—Terminology Consensus Project process and outcome. Int. J. Behav. Nutr. Phys. Act..

[45] Malik P, Patel K, Pinto C, Jaiswal R, Tirupathi R, Pillai S (2022). Post-acute COVID-19 syndrome (PCS) and health-related quality of life (HRQoL)-A systematic review and meta-analysis. J. Med. Virol..

[46] Gomez-Moreno SM, Cuadrado ML, Cruz-Orduña I, Martínez-Acebes EM, Gordo-Mañas R, Fernández-Pérez C (2021). Validation of the Spanish-language version of the Montreal Cognitive Assessment as a screening test for cognitive impairment in multiple sclerosis. Neurologia (Barcelona, Spain).

[47] Tabacof L, Tosto-Mancuso J, Wood J, Cortes M, Kontorovich A, McCarthy D (2022). Post-acute COVID-19 syndrome negatively impacts physical function, cognitive function, health-related quality of life, and participation. Am. J. Phys. Med. Rehabil..

[48] Poudel AN, Zhu S, Cooper N, Roderick P, Alwan N, Tarrant C (2021). Impact of Covid-19 on health-related quality of life of patients: A structured review. PloS One.

[49] Koopmans PC, Bakhtali R, Katan AA, Groothoff JW, Roelen CA (2010). Return to work following sickness absence due to infectious mononucleosis. Occup. Med..

[50] Twomey R, Demars J, Franklin K, Nicole Culos-Reed S, Weatherald J, Wrightson JG (2022). Chronic fatigue and postexertional malaise in people living with long COVID: An observational study. Phys. Ther..

[51] Fernández-Lázaro D, Santamaría G, Sánchez-Serrano N, Lantarón Caeiro E, Seco-Calvo J (2022). Efficacy of therapeutic exercise in reversing decreased strength, impaired respiratory function, decreased physical fitness, and decreased Quality of life caused by the post-COVID-19 syndrome. Viruses.

[52] Boutou AK, Asimakos A, Kortianou E, Vogiatzis I, Tzouvelekis A (2021). Long COVID-19 pulmonary sequelae and management considerations. J. Personal. Med..

[53] Aly MAEG, Saber HG (2021). Long COVID and chronic fatigue syndrome: A survey of elderly female survivors in Egypt. Int. J. Clin. Pract..

[54] Cunningham C, O’Sullivan R, Caserotti P, Tully MA (2020). Consequences of physical inactivity in older adults: A systematic review of reviews and meta-analyses. Scand. J. Med. Sci. Sports..

[55] Lund LC, Hallas J, Nielsen H, Koch A, Mogensen SH, Brun NC (2021). Post-acute effects of SARS-CoV-2 infection in individuals not requiring hospital admission: A Danish population-based cohort study. Lancet Infect. Dis..

