# Supplementary material for: Functionality, physical activity, fatigue and quality of life in patients with acute COVID-19 and Long COVID infection
Source: Sci Rep. 2023 Nov 14;13:19907. doi: 10.1038/s41598-023-47218-1 (PMC10645778; doi:10.1038/s41598-023-47218-1)
Supplement: Supplementary file 1 — Supplementary Tables. [file 41598_2023_47218_MOESM1_ESM.docx]

**Supplementary material**

Supplementary table 1. Correlation between Functional status post-COVID-19 and group

|  | | Functional status post-COVID-19 | | | | | Total |
| --- | --- | --- | --- | --- | --- | --- | --- |
|  |  | No functional limitations | Non-significant functional limitation | Mild functional limitation | Moderate functional limitation | Severe functional limitation |  |
| Group 1 | Count | 1 | 1 | 6 | 20 | 57 | 85 |
|  | Expected Count | 29.5 | 4.0 | 6.5 | 15.0 | 30.0 | 85.0 |
|  | % within group | 1.2% | 1.2% | 7.1% | 23.5% | 67.1% | 100.0% |
|  | % within functional status post-COVID-19 | 1.7% | 12.5% | 46.2% | 66.7% | 95.0% | 50,0% |
|  | % total | 0.6% | 0.6% | 3.5% | 11.8% | 33.5% | 50.0% |
|  | Corrected residue | -9.2 | -2.2 | -0.3 | 2.0 | 8.7 |  |
| Group 2 | Count | 58 | 7 | 7 | 10 | 3 | 85 |
|  | Expected Count | 29.5 | 4.0 | 6.5 | 15.0 | 30.0 | 85.0 |
|  | % within group | 68.2% | 8.2% | 8.2% | 11.8% | 3.5% | 100.0% |
|  | % within functional status post-COVID-19 | 98.3% | 87.5% | 53.8% | 33.3% | 5.0% | 50.0% |
|  | % total | 34.1% | 4.1% | 4.1% | 5.9% | 1.8% | 50.0% |
|  | Corrected residue | 9.2 | 2.2 | 0.3 | -2.0 | -8.7 |  |
| Total | Count | 59 | 8 | 13 | 30 | 60 | 170 |
|  | Expected Count | 59.0 | 8.0 | 13.0 | 30.0 | 60.0 | 170.0 |
|  | % within group | 34.7% | 4.7% | 7.6% | 17.6% | 35.3% | 100.0% |
|  | % within functional status post-COVID-19 | 100.0% | 100.0% | 100.0% | 100.0% | 100.0% | 100.0% |
|  | % total | 34.7% | 4.7% | 7.6% | 17.6% | 35.3% | 100.0% |

Supplementary table 2. Correlation between level of physical activity pre-COVID-19 and current, and group

|  | | Current level of physical activity | | | Total |
| --- | --- | --- | --- | --- | --- |
|  |  | Low | Moderate | High |  |
| Group 1 | Count | 3 | 26 | 56 | 85 |
|  | Expected Count | 5.5 | 33.0 | 46.5 | 85.0 |
|  | % within group | 3.5% | 30.6% | 65.9% | 100.0% |
|  | % within current level of physical activity | 27.3% | 39.4% | 60.2% | 50.0% |
|  | % total | 1.8% | 15.3% | 32.9% | 50.0% |
|  | Corrected residue | -1.6 | -2.2 | 2.9 |  |
| Group 2 | Count | 8 | 40 | 37 | 85 |
|  | Expected Count | 5.5 | 33.0 | 46.5 | 85.0 |
|  | % within group | 9.4% | 47.1% | 43.5% | 100.0% |
|  | % within current level of physical activity | 72.7% | 60.6% | 39.8% | 50.0% |
|  | % total | 4.7% | 23.5% | 21.8% | 50.0% |
|  | Corrected residue | 1.6 | 2.2 | -2.9 |  |
| Total | Count | 11 | 66 | 93 | 170 |
|  | Expected Count | 11.0 | 66.0 | 93.0 | 170.0 |
|  | % within current level of physical activity | 100.0% | 100.0% | 100.0% | 100.0% |

Supplementary table 3. Correlation between current level of physical activity and group

|  | | Level of physical activity pre-COVID-19 and current | | | Total |
| --- | --- | --- | --- | --- | --- |
|  |  | Low | Moderate | High |  |
| Group 1 | Count | 23 | 43 | 19 | 85 |
|  | Expected Count | 14.5 | 43.0 | 27.5 | 85.0 |
|  | % within group | 27.1% | 50.6% | 22.4% | 100.0% |
|  | % within level of physical activity pre-COVID-19 and current | 79.3% | 50.0% | 34.5% | 50.0% |
|  | % total | 13.5% | 25.3% | 11.2% | 50.0% |
|  | Corrected residue | 3.5 | 0.0 | -2.8 |  |
| Group 2 | Count | 6 | 43 | 36 | 85 |
|  | Expected Count | 14.5 | 43.0 | 27.5 | 85.0 |
|  | % within group | 7.1% | 50.6% | 42.4% | 100.0% |
|  | % within level of physical activity pre-COVID-19 and current | 20.7% | 50.0% | 65.5% | 50.0% |
|  | % total | 3.5% | 25.3% | 21.2% | 50.0% |
|  | Corrected residue | -3.5 | 0.0 | 2.8 |  |
| Total | Count | 29 | 86 | 55 | 170 |
|  | Expected Count | 29.0 | 86.0 | 55.0 | 170.0 |
|  | % within group | 17.1% | 50.6% | 32.4% | 100.0% |
|  | % within level of physical activity pre-COVID-19 and current | 100.0% | 100.0% | 100.0% | 100.0% |
|  | % total | 17.1% | 50.6% | 32.4% | 100.0% |

Supplementary table 4. Correlation between Variation in physical activity level pre COVID and current, and group

|  | | Variation in physical activity level pre COVID and current | | | | | Total |
| --- | --- | --- | --- | --- | --- | --- | --- |
|  |  | It has worsened significantly | It has worsened slightly | It has remained the same | It has improved slightly | It has improved significantly |  |
| Group 1 | Count | 12 | 38 | 30 | 5 | 0 | 85 |
|  | Expected Count | 6.0 | 22.5 | 51.0 | 4.5 | 1.0 | 85.0 |
|  | % within group | 14.1% | 44.7% | 35.3% | 5.9% | 0.0% | 100.0% |
|  | % within variation in physical activity level pre COVID and current | 100.0% | 84.4% | 29.4% | 55.6% | 0.0% | 50.0% |
|  | % total | 7.1% | 22.4% | 17.6% | 2.9% | 0.0% | 50.0% |
|  | Corrected residue | 3.6 | 5.4 | -6.6 | 0.3 | -1.4 |  |
| Group 2 | Count | 0 | 7 | 72 | 4 | 2 | 85 |
|  | Expected Count | 6.0 | 22.5 | 51.0 | 4.5 | 1.0 | 85.0 |
|  | % within group | 0.0% | 8.2% | 84.7% | 4.7% | 2.4% | 100.0% |
|  | % within variation in physical activity level pre COVID and current | 0.0% | 15.6% | 70.6% | 44.4% | 100.0% | 50.0% |
|  | % total | 0.0% | 4.1% | 42.4% | 2.4% | 1.2% | 50.0% |
|  | Corrected residue | -3.6 | -5.4 | 6.6 | -0.3 | 1.4 |  |
| Total | Count | 12 | 45 | 102 | 9 | 2 | 170 |
|  | Expected Count | 12.0 | 45.0 | 102.0 | 9.0 | 2.0 | 170.0 |
|  | % within group | 7.1% | 26.5% | 60.0% | 5.3% | 1.2% | 100.0% |
|  | % within variation in physical activity level pre COVID and current | 100.0% | 100.0% | 100.0% | 100.0% | 100.0% | 100.0% |
|  | % total | 7.1% | 26.5% | 60.0% | 5.3% | 1.2% | 100.0% |

Supplementary table 5. Correlation between QoL pre COVID-19 and group

|  | | QoL pre COVID-19 | | | | Total |
| --- | --- | --- | --- | --- | --- | --- |
|  |  | Excellent | Very good | Good | Fair |  |
| Group 1 | Count | 14 | 39 | 29 | 3 | 85 |
|  | Expected Count | 13.0 | 31.0 | 34.5 | 6.5 | 85.0 |
|  | % within group | 16.5% | 45.9% | 34.1% | 3.5% | 100.0% |
|  | % within QoL pre COVID-19 | 53.8% | 62.9% | 42.0% | 23.1% | 50.0% |
|  | Corrected residue | 0.4 | 2.5 | -1.7 | -2.0 |  |
| Group 2 | Count | 12 | 23 | 40 | 10 | 85 |
|  | Expected Count | 13.0 | 31.0 | 34.5 | 6.5 | 85.0 |
|  | % within group | 14.1% | 27.1% | 47.1% | 11.8% | 100.0% |
|  | % within QoL pre COVID-19 | 46.2% | 37.1% | 58.0% | 76.9% | 50.0% |
|  | Corrected residue | -0.4 | -2.5 | 1.7 | 2.0 |  |
| Total | Count | 26 | 62 | 69 | 13 | 170 |
|  | Expected Count | 26.0 | 62.0 | 69.0 | 13.0 | 170.0 |
|  | % within group | 15.3% | 36.5% | 40.6% | 7.6% | 100.0% |
|  | % within QoL pre COVID-19 | 100.0% | 100.0% | 100.0% | 100.0% | 100.0% |

Supplementary table 6. Correlation between current QoL and group

|  | | Current Qol | | | | | Total |
| --- | --- | --- | --- | --- | --- | --- | --- |
|  |  | Excellent | Very good | Good | Fair | Poor |  |
| Group 1 | Count | 1 | 0 | 9 | 37 | 38 | 85 |
|  | Expected Count | 6.0 | 10.0 | 24.0 | 25.0 | 20.0 | 85.0 |
|  | % within group | 1.2% | 0.0% | 10.6% | 43.5% | 44.7% | 100.0% |
|  | % within current Qol | 8.3% | 0.0% | 18.8% | 74.0% | 95.0% | 50.0% |
|  | % total | 0.6% | 0.0% | 5.3% | 21.8% | 22.4% | 50.0% |
|  | Corrected residue | -3.0 | -4.8 | -5.1 | 4.0 | 6.5 |  |
| Group 2 | Count | 11 | 20 | 39 | 13 | 2 | 85 |
|  | Expected Count | 6.0 | 10.0 | 24.0 | 25.0 | 20.0 | 85.0 |
|  | % within group | 12.9% | 23.5% | 45.9% | 15.3% | 2.4% | 100.0% |
|  | % within current Qol | 91.7% | 100.0% | 81.3% | 26.0% | 5.0% | 50.0% |
|  | % total | 6.5% | 11.8% | 22.9% | 7.6% | 1.2% | 50.0% |
|  | Corrected residue | 3.0 | 4.8 | 5.1 | -4.0 | -6.5 |  |
| Total | Count | 12 | 20 | 48 | 50 | 40 | 170 |
|  | Expected Count | 12.0 | 20.0 | 48.0 | 50.0 | 40.0 | 170.0 |
|  | % within group | 7.1% | 11.8% | 28.2% | 29.4% | 23.5% | 100.0% |
|  | % within current Qol | 100.0% | 100.0% | 100.0% | 100.0% | 100.0% | 100.0% |
|  | % total | 7.1% | 11.8% | 28.2% | 29.4% | 23.5% | 100.0% |

Supplementary table 7. Correlation between variation in QoL pre COVID and current

|  | | Variation in QoL pre COVID and current | | | | | | Total |
| --- | --- | --- | --- | --- | --- | --- | --- | --- |
|  |  | It has worsened significantly | It has worsened slightly | It has remained the same | It has improved slightly | It has improved significantly | It has worsened significantly |  |
| Group 1 | Count | 3 | 18 | 46 | 17 | 1 | 0 | 85 |
|  | Expected Count | 1.5 | 9.0 | 25.0 | 11.0 | 38.0 | 0.5 | 85.0 |
|  | % within group | 3.5% | 21.2% | 54.1% | 20.0% | 1.2% | 0.0% | 100.0% |
|  | % within variation in QoL pre COVID and current | 100.0% | 100.0% | 92.0% | 77.3% | 1.3% | 0.0% | 50.0% |
|  | % total | 1.8% | 10.6% | 27.1% | 10.0% | 0.6% | 0.0% | 50.0% |
|  | Corrected residue | 1.7 | 4.5 | 7.1 | 2.7 | -11.4 | -1.0 |  |
| Group 2 | Count | 0 | 0 | 4 | 5 | 75 | 1 | 85 |
|  | Expected Count | 1.5 | 9.0 | 25.0 | 11.0 | 38.0 | 0.5 | 85.0 |
|  | % within group | 0.0% | 0.0% | 4.7% | 5.9% | 88.2% | 1.2% | 100.0% |
|  | % within variation in QoL pre COVID and current | 0.0% | 0.0% | 8.0% | 22.7% | 98.7% | 100.0% | 50.0% |
|  | % total | 0.0% | 0.0% | 2.4% | 2.9% | 44.1% | 0.6% | 50.0% |
|  | Corrected residue | -1.7 | -4.5 | -7.1 | -2.7 | 11.4 | 1.0 |  |
| Total | Count | 3 | 18 | 50 | 22 | 76 | 1 | 170 |
|  | Expected Count | 3.0 | 18.0 | 50.0 | 22.0 | 76.0 | 1.0 | 170.0 |
|  | % within group | 1.8% | 10.6% | 29.4% | 12.9% | 44.7% | 0.6% | 100.0% |
|  | % within variation in QoL pre COVID and current | 100.0% | 100.0% | 100.0% | 100.0% | 100.0% | 100.0% | 100.0% |
|  | % total | 1.8% | 10.6% | 29.4% | 12.9% | 44.7% | 0.6% | 100.0% |

Supplementary table 8. Correlation between variation in level of fatigue pre COVID and current, and group

|  | | Variation in level of fatigue pre COVID and current | | | | | | Total |
| --- | --- | --- | --- | --- | --- | --- | --- | --- |
|  |  | It has worsened significantly | It has worsened slightly | It has remained the same | It has improved slightly | It has improved significantly | No fatigue before and now |  |
| Group 1 | Count | 18 | 4 | 25 | 19 | 19 | 0 | 85 |
|  | Expected Count | 9.5 | 6.0 | 24.0 | 10.5 | 10.5 | 24.5 | 85.0 |
|  | % within group | 21.2% | 4.7% | 29.4% | 22.4% | 22.4% | 0.0% | 100.0% |
|  | % within variation in level of fatigue pre COVID and current | 94.7% | 33.3% | 52.1% | 90.5% | 90.5% | 0.0% | 50.0% |
|  | % total | 10.6% | 2.4% | 14.7% | 11.2% | 11.2% | 0.0% | 50.0% |
|  |  | 4.1 | -1.2 | 0.3 | 4.0 | 4.0 | -8.3 |  |
| Group 2 | Count | 1 | 8 | 23 | 2 | 2 | 49 | 85 |
|  | Expected Count | 9.5 | 6.0 | 24.0 | 10.5 | 10.5 | 24.5 | 85.0 |
|  | % within group | 1.2% | 9.4% | 27.1% | 2.4% | 2.4% | 57.6% | 100.0% |
|  | % within variation in level of fatigue pre COVID and current | 5.3% | 66.7% | 47.9% | 9.5% | 9.5% | 100.0% | 50.0% |
|  | % total | 0.6% | 4.7% | 13.5% | 1.2% | 1.2% | 28.8% | 50.0% |
|  | Corrected residue | -4.1 | 1.2 | -0.3 | -4.0 | -4.0 | 8.3 |  |
| Total | Count | 19 | 12 | 48 | 21 | 21 | 49 | 170 |
|  | Expected Count | 19.0 | 12.0 | 48.0 | 21.0 | 21.0 | 49.0 | 170.0 |
|  | % within group | 11.2% | 7.1% | 28.2% | 12.4% | 12.4% | 28.8% | 100.0% |
|  | % within variation in level of fatigue pre COVID and current | 100.0% | 100.0% | 100.0% | 100.0% | 100.0% | 100.0% | 100.0% |
|  | % total | 11.2% | 7.1% | 28.2% | 12.4% | 12.4% | 28.8% | 100.0% |

Supplementary table 9. Correlation between SFC and group

|  | | SFC (compatible yes/no) | | Total |
| --- | --- | --- | --- | --- |
|  |  | Yes | No |  |
| Group 1 | Count | 73 | 11 | 84 |
|  | Expected Count | 39.3 | 44.7 | 84.0 |
|  | % within group | 86.9% | 13.1% | 100.0% |
|  | % within SFC (compatible yes/no) | 92.4% | 12.2% | 49.7% |
|  | % total | 43.2% | 6.5% | 49.7% |
|  | Corrected residue | 10.4 | -10.4 |  |
| Group 2 | Count | 6 | 79 | 85 |
|  | Expected Count | 39.7 | 45.3 | 85.0 |
|  | % within group | 7.1% | 92.9% | 100.0% |
|  | % within SFC (compatible yes/no) | 7.6% | 87.8% | 50.3% |
|  | % total | 3.6% | 46.7% | 50.3% |
|  | Corrected residue | -10.4 | 10.4 |  |
| Total | Count | 79 | 90 | 169 |
|  | Expected Count | 79.0 | 90.0 | 169.0 |
|  | % within group | 46.7% | 53.3% | 100.0% |
|  | % within SFC (compatible yes/no) | 100.0% | 100.0% | 100.0% |
|  | % total | 46.7% | 53.3% | 100.0% |
